# The expression and clinical significance of TPM4 in hepatocellular carcinoma

**DOI:** 10.7150/ijms.49906

**Published:** 2021-01-01

**Authors:** Linjing Li, Tao Ye, Qingyan Zhang, Xin Li, Li Ma, Jing Yan

**Affiliations:** 1 Department of Clinical Laboratory Center, The Second Hospital of Lanzhou University, Lanzhou 730000, P.R. China.; 2 The First School of Clinical Medicine, Southern Medical University, Guangzhou, China, 510515.; 3 Center of Reproductive Medicine, Department of Obstetrics and Gynecology, First Affiliated Hospital of Sun Yat-sen University, Guangzhou, China, 510080.

**Keywords:** hepatocellular carcinoma, TPM4, biological marker, clinicopathological parameters, diagnosis

## Abstract

Hepatocellular carcinoma (HCC) is known as the fifth most common cancer in the world for its poor prognosis. New diagnostic markers and treatments are urgent to discover. To evaluate the protein expression of Tropomyosin4 (TPM4) and investigate its prognostic value in HCC, we collected 110 patients with different degrees of HCC and 10 patients with normal hepatic tissues and performed immunohistochemistry. Western bot was used to evaluate the expression of TPM4 in three HCC cell lines (HepG2, Huh7, SMMC-7721) and normal liver cell line LO2, as well as 7 HCC tissues and 7 normal hepatic tissues. The results of TPM4 staining revealed that TPM4 expression in HCC was higher than that in normal hepatic tissues, which was positive in 51.8% (n=57) and negative in 48.2% (n=53) while in normal hepatic tissues positive staining was in 10% (n=1) and negative staining was in 90% (n=9) (*P*=0.011). And the expression of TPM4 was related to pT status, grade and stage (*P*<0.001, *P*=0.015 and *P*<0.001, respectively). Western blot results indicated that TPM4 was high expressed in HCC cell line and HCC tissues. In conclusion, we believe that TPM4 can be applied as a diagnostic and prognostic marker to assist the management of HCC.

## Introduction

Liver cancer can be classified as primary and secondary. According to the statistics, liver cancer is the dominating cause of cancer mortality in males of Southern China 1. It occupies the fifth position in tumor and ranks fourth among cancer subtypes, and accounts for more than 50% of the world's total in China 1,2. Primary liver cancer is considered as one of the most aggressive cancers. Its occurrence is a multifactorial and complex progress with poor prognosis. Moreover, many patients are insensitive to the relevant drugs during the treatment. The majority of primary liver cancer is hepatocellular carcinoma (HCC), which is always diagnosed in the advanced stage 3. From 2007 to 2016, the incidence of liver cancer increased rapidly at the rate of 2% to 3% 4. The dominating inducement of HCC is the chronic infection based on hepatitis B virus (HBV) and hepatitis C virus (HCV), and the latter was rarely detected 4,5. No matter what kind of liver cancer it is, operation will be the most appropriate surgical treatment in the early stage and radiofrequency ablation will be used in the last stage. However, chemical treatments are not recommended for its low efficiency 2. At present, some biomarkers can be applied to analyze the prognosis of HCC, for instance, the significant advantage of albumin-bilirubin grade in HCC and other liver diseases have been widely acknowledged 6. In spite of this, some biological markers which are able to assist diagnosis need to be discovered. With the advance of modern medicine, the situation could be improved.

Tropomyosin4 (TPM4) belongs to the tropomyosin family, member of which are actin-binding proteins. It helps regulate actomyosin interaction in muscle by extending α-helical coiled-coil homodimer 7. TPM4 is expressed in blood platelet and has an effect on the volume and number of platelets. It has been confirmed that TPM4 is the independent risk factor of stroke or heart diseases 8, while some specific tumors in human bodies may express abnormal levels of TPM4. Therefore, TPM4 is likely to be used as a marker for several cancers. By changing actin cytoskeleton, TPM4 enhances the migration of tumor cells in lung cancer 9. High expression of TPM4 in ovarian tumor tissue was first reported in 2004 10. Detecting the expression of proteins TPM4, ANXA6, HSP27, PRDX2 and NCF2 may contribute to the pathological and cytological diagnosis and prognosis assessment in squamous cervical cancer 11. However, there are few cases of diagnosing HCC by testing the content of TPM4. Therefore, it's highly desirable to explore the connection between the expression of TPM4 and HCC, including its TNM classification, so that it may provide reference information for the future medicine.

## Materials and Methods

### Cell lines and cultures

Human hepatic-derived hepatoma G2 cell (HepG2) was purchased from American Type Culture Collection (ATCC HB-8065, Rockville, MD, USA) and it was cultivated in Dulbecco's Modified Eagle Medium (DMEM, Gibco, USA), supplemented with 10% fetal bovine serum (FBS, Gibco, Life Technologies, Melbourne, Australia). Human HCC cell line Huh7 was saved in laboratory and maintained in DMEM added 10% FBS, 10 mM HEPES, 100 U/ml of penicillin, and 100 μg/ml streptomycin. Human HCC cell line SMMC-7721 was preserved in laboratory and cultured in RMPI 1640 (1640, Gibco, USA) which contained 10% FBS. Human normal cells LO2 was obtained from laboratory conservation, maintained in DMEM, 10% FBS. All cells were maintained in a humidified incubator at 37°C with 5% CO_2_.

### Tissue samples

We have gathered the information of patients with hepatoma from Second affiliated hospital of Lanzhou university between 2015 and 2019. There were 120 pathological sections of hepatic in total, among which there were 110 samples from patients with hepatoma without treatment and the remaining 10 samples were from normal tissues. Other information included age, sex, pathology diagnosis, histology grade, clinical stage and pTNM. The mean age was 49.1 and the rest details were demonstrated in **Table 2.** The study obeyed the protocol of the Ethic Committee of Second affiliated hospital of Lanzhou university and written informed consents were also obtained.

### Western Blot

In the presence of the protease inhibitors, total protein was isolated from HCC cells by Radio-Immunoprecipitation Assay (RIPA) buffer (Beyo-time, China), which contained 1mM PMSF (Beyo-time, China). The protein concentration was then measured using BCA Protein Assay Kit (Beyo-time, China) and normalized using the standard BSA curve. These samples were dissolved in loading buffer and unstructured for 10 mins by boiling. In the end, equivalent amounts of proteins were separated by 10% SDS-PAGE gel electrophoresis and transferred to polyvinylidene fluoride (PVDF) membrane (Millipore, USA) on a standard wet-transfer equipment (Trans-blot SD, Bio-Rad, USA). The membranes were then blocked in Tris-Buffer Saline Tween 20 (1×TBST) containing 5% non-fat milk at room temperature. After 2 hours, membranes would be incubated with TPM4 (rabbit polyclonal, 1:1000, Proteintech, China) primary antibody and GAPDH primary antibody (Mouse monoclonal, 1:2000, Proteintech, China) at 4°C for a night. After three times washing with 1×TBST for 10 mins, we again incubated the membranes with secondary antibodies labeled with horseradish peroxidase-(HRP) (Go-at anti-Rabbit IgG, 1:5000, MultiSciences, China; Goat anti-Mouse IgG, 1:5000, MultiSciences, China) at room temperature for 1.5 hours. Afterwards, the membranes were then washed with 1×TBST for 3 times, followed by the enhanced chemiluminescence reagents (BeyoECL Plus Kit, Beyotime, China), data scanning and protein bands were recorded by Tanon-5200 (Tanon, Shanghai, China). The optical density of protein bands was quantitatively determined.

### Tissue microarrays (TMA) construction and immunohistochemistry

The construction of TMA covered 110 pathological sections of HCC and 10 normal samples according to the standard method 12. Continuous 5 μm portion of pathological section was removed in TMA block mass for immunohistochemical analysis, followed by staining basing on the standard procedures. Subsequently, the slices were dewaxed in xylene and rehydrated in fractional ethanol. Activity of endogenous catalase would be inhibited by 0.3% H_2_O_2_. After antigen recovery, the citrate buffer was heated with sodium for 20 mins in a self-cleavage tank for 20 mins. Sections were then incubated and 1×phosphate-buffered saline (PBS) which contained 5% normal goat serum for blocking was used for 30 mins. Sections were cultured at 4°C with primary antibody from TPM4 (rabbit polyclonal, Proteintech, China) for a night, following being washed for 3 times by 1×PBS and cultivated with HRP conjugated-secondary antibodies at room temperature. 1×PBS would be applied for additional wash after 30 mins. Under the use of hematoxylin and dehydrated before mounting, these sections were stained. Immunostaining was performed by DAB Horseradish Peroxidase Color Development Kit (Beyotime, China). Phosphate buffered saline took the place of anti-TPM4 antibody to act as a negative control.

### IHC Evaluation

There were two authors who accounted for the immunostaining results of TPM4 on a semi-quantitative scale of staining intensity and percentage. They did not possess the prior knowledge about the characteristics of the patients. Cytoplasmic staining was measured as the positive immunostaining. Percentage fractions were 5 increments (0, 5%, 10%, ..., 100%). Only when the differences were harmonized did the two authors stop re-estimating.

### Selection of cut-off score

The optimal cut-off score was obtained through Receiver operating characteristic (ROC) curve analysis, and the 0, 1-criterion was created by referring to the incremental expression of TPM4 13. We divided the clinicopathological parameters analyzed by ROC into two groups: histological grade (G0-G2, G3), pT stage (T0-T2, T3-T4), clinical stage (I-II, III-IV). Subsequently, the ROC curves were drawn based on the specificity and sensitivity of each TPM4 score. The point closest to the given point [0.0, 1.0] would be considered as the cut-off score. Therefore, the tumor would be regarded as TPM4 “negative” if the last score had been below the “cut-off score”. Otherwise, the tumor would be regarded as TPM4 “positive”.

### Statistical analysis

SPSS 20.0 software (SPSS, Chicago, IL, USA) was used to complete statistical estimations. Data was expressed as mean ± standard deviation with no less than three independent experiments included. Optimal cut-off point of TPM4 positive was obtained by ROC being introduced. Applied T-test was used to search for expression distinction between two groups. The chi-square served as a role to evaluate the connection between TPM4 expression and the clinicopathological parameters of hepatoma. If *P* values were less than 0.05, a distinction would be made as statistically significant.

## Results

### TPM4 expression in HCC cells

**Figure 1:**
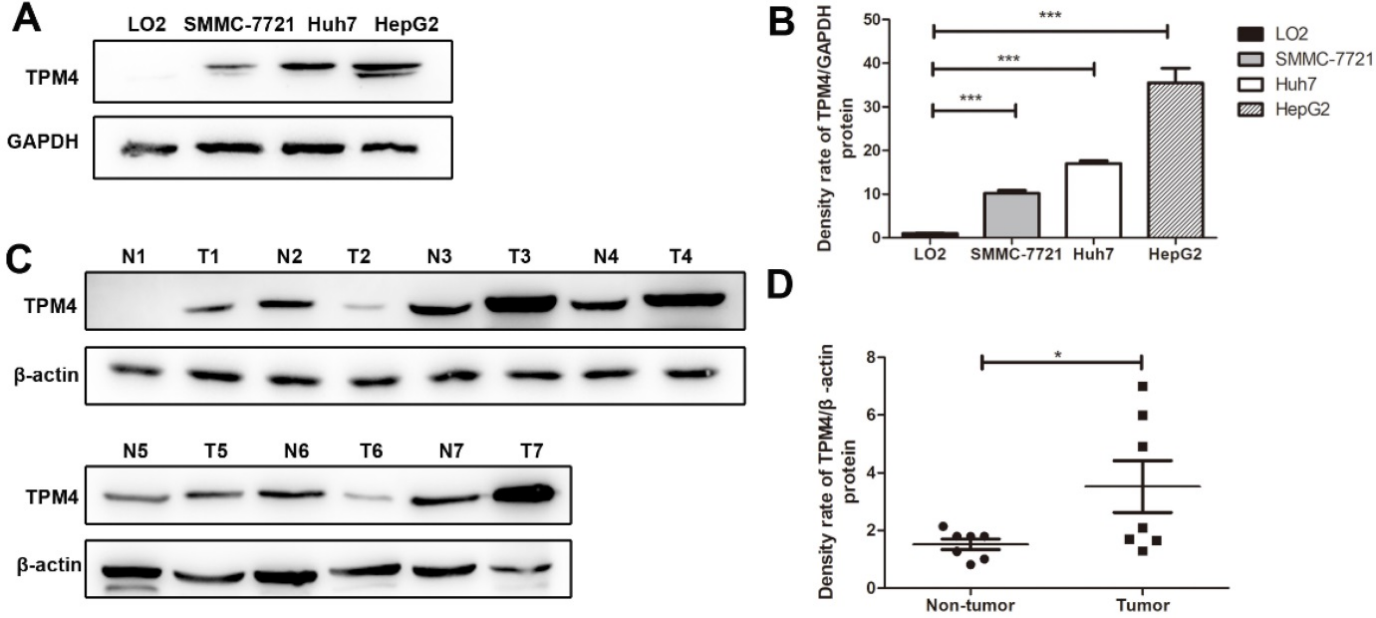
** Western blot results of TPM4 expression in liver cell lines and tissues. A.** Western blot indicated significant up-regulation in hepatocellular carcinoma cells (SMMC-7721, Huh7, HepG2) compared to in the human normal immortalized cell lines (LO2). GAPDH was used as control. **C.** Western blot results of TPM4 in 7 hepatocellular carcinoma tissues(T1-T7) and 7 normal hepatocellular tissues(N1-N7) **B.D.** Western blot results were measured as optical density value and expressed graphically. TPM4 protein expressions were significantly higher in hepatocellular carcinoma cells and tissues (*P<0.05, **P<0.01, ***P<0.001).

Through western blot, we successfully examined the expression of TPM4 in three human HCC cell lines, HepG2, Huh7, SMMC-7721 and human normal immortalized liver cell line, LO2. The results showed that TPM4 expression in HCC cell lines were distinctly higher than that in human normal liver cell line (**Figure 1**).

### TPM4 expression in tissue samples of HCC

**Figure 2:**
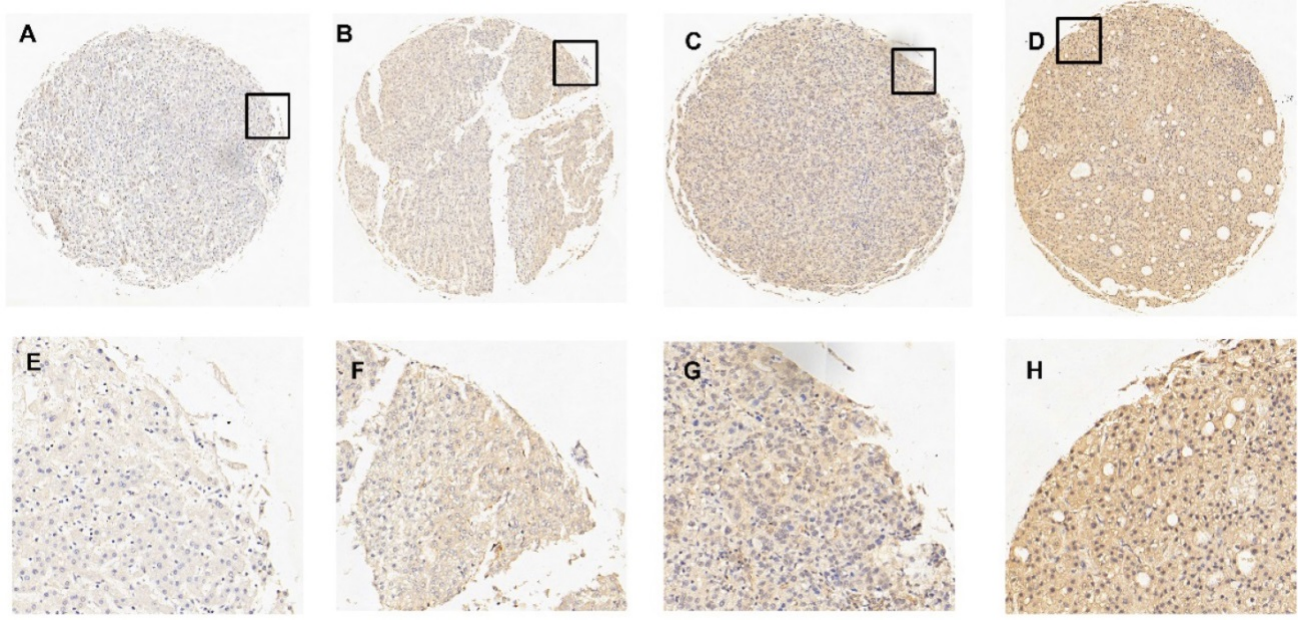
** IHC results of the expression of TPM4 in normal hepatic tissues and hepatocellular carcinoma tissues. A.** 5% positive expression of TPM4 was detected in hepatic normal tissue (case 117) (10×). **B.** 40% positive expression of TPM4 was detected in a hepatocellular carcinoma tissue (case 80) (10×). **C.** 80% positive expression of TPM4 was showed in a hepatocellular carcinoma tissue (case 45) (10×). **D.** 95% positive expression of TPM4 was showed in hepatocellular carcinoma (case 61) (10×). **E-H.** Demonstrate the higher magnification (40×) from the area of black box in (A-D), respectively.

**Figure 3:**
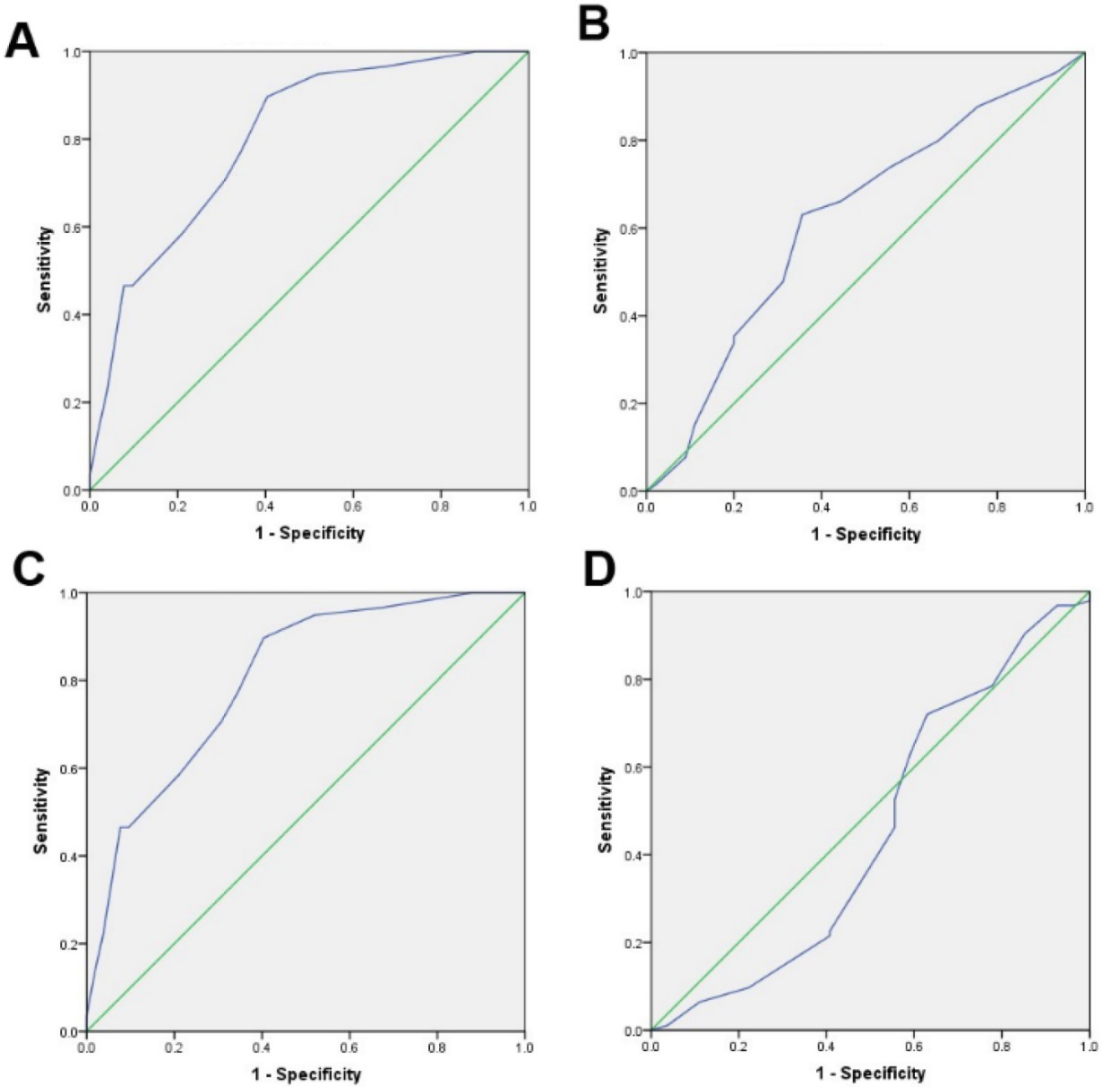
** Receiver Operating Characteristic (ROC) was used to select the optimal cut-off score for positive expression of TPM4: A.** pT stage. **B.** Histological grade. **C.** Clinical stage. **D.** Sex. The cut-off score is 67.5%.

Compared with normal hepatic tissues, we evaluated the content of TPM4 in HCC tissues by applying immunohistochemistry (IHC). Immunoreactivity could be detected in the cell membrane of tumor cells. **Figure 2** has shown 4 typical samples of different levels of TPM4 IHC staining. The optimal cut-off score was determined by the analysis from Receiver operating Characteristic curve (ROC). **Figure 3** showed that the clinical stage and pT status were both chosen for the shortest distance from point (0.0, 1.0). Therefore, 67.5% would be considered as the optimal cut-off score, above which was supposed to be positive and otherwise was supposed to be negative. According to the cut-off score, 51.8% (n=57) remained to be positive and the rest 48.2% (n=53) remained to be negative after TPM4 staining. In normal hepatic tissues with TPM4 staining, 10% (n=1) of normal cases suggested to be positive while 90% (n=9) suggested to be negative. Using Chi-square test for further analysis, we tried to explore the connection between the expression of TPM4 and HCC and normal hepatic tissue which showed that TPM4 expression in HCC was distinctly higher than that in human normal hepatocytes (χ^2^=6.419 *P*=0.011) (**Table 1**).

### Correlation between TPM4 and clinicpathological parameters

In addition, we separately analyzed the connection between TPM4 expression and clinicopathological parameters of HCC cell lines. **Table 2** indicated that TPM4 positive was related to pT status, histological grade and clinical stage (all* P <* 0.05). While, there was not enough evidence to prove that sex and age were correlated to TPM4 positive (all *P >* 0.05).

## Discussion

HCC is acknowledged as a serious kind of cancer, which is recognized around the world. Currently, major breakthroughs have been made not only in the treatment of HCC but also in the prediction of tumor recurrence after clinical treatment 14. TPM4 is involved in the regulation of actin binding proteins, and TPM4 was reported that it related the progress in several cancers. It was reported that TPM4 was highly detected in the serum of ovarian patients. And TPM4 may act as an oncogene and a potential early diagnosis marker in breast cancer, lung cancer and esophageal squamous cell carcinoma 15. However, the relationship of TPM4 and HCC is unknown.

To reveal the expression pattern of TPM4 in cells from HCC, our experiment was conducted on three HCC cells lines (HepG2, Huh7, SMMC-7721) and one normal hepatic immortalized human urothelial cell line (LO2) by applying western blot. The results of the experiments indicated that the content of TPM4 in three HCC cell lines were up-regulated apparently compared with the normal hepatic immortalized human urothelial cell line (*P <* 0.05). Cell lines are models *in vitro* and can explain the expression of TPM4 in some degree. We hypothesized that TPM4 was highly expressed in HCC. Futhermore, 7 pairs of HCC tissues and normal hepatic tissues were used to verify the hypothesis. The results indicated that TPM4 was high expressed in HCC (*P <* 0.05).

However, the role of TPM4 in the diagnosis of HCC is still unclear. To evaluate the clinical application value of TPM4, there were 110 hepatoma tissues and 10 normal hepatic tissues collected and they were constructed TMA stained with TPM4. Subsequently, we obtained the optimal cut-off score by adopting ROC curve. Basing on 4 clinicopathological parameters including histological grade, clinical stage, pT stage and sex, ROC curves were then created. “67.5%” was defined as the optimal cut-off score, staining above which would be regarded as “TPM4” positive. Statistic result represented that expression of TPM4 in HCC tissues were higher than that in normal hepatic tissues. More detailed analysis, higher expression of TPM4 was correlated with histological grade, clinical stage, pT stage but had nothing to do with sex and age. In the study, we found that the expression of TPM4 will increase with the aggravation of histological grade, pT and clinical stage while the content is low in the normal hepatic cells.

Many studies focused on identifying the candidate biomarkers for HCC, such as genetic mutations, gene expression and non-coding RNAs. Alpha-fetoprotein (AFP) is a widely used marker for detecting and monitoring HCC, however, AFP serum levels stay normal in nearly 30% of advanced HCC patients 16. Other markers have been reported, but most performed an unsatisfactory sensitivity or specificity. There are experiments which can prove that the progression of HCC can be inhibited by raising the expression of three main genes which are cysteine sulfinic acid decarboxylase (CSAD), glutamic-oxaloacetic transaminase 2 (GOT2) and suppressor of cytokine signaling 2 (SOCS2) through METTL14 17,18,19. Ribosomal protein S11 (RPS11) overexpression is affiliated with variety of malignancies and tumor recurrence, which can be used as a potential biomarker for predicting the prognosis of patients who have received radical resection of HCC 20. Besides, studies suggested that p38γ is able to interact with CDKs acting as a type of CDK-like-kinase and thus regulating the cell cycle. And high expression of p38γ can be detected in human HCC and might be considered as a therapeutic target 21. JNK pathway plays an initial role in maintaining hepatic homeostasis. Numerous diseases are correlated with its dysregulation, including steatosis, fibrosis, cirrhosis and hepatoma 22, 23, 24. The abnormality or deficiency of JNK will inhibit the proliferation of HCC cells 25, 26, 27. However, the factors related to the pathogenesis have not been completely identified because of its complicated mechanism, and the roles of TPM4 in HCC need to be further studied.

Finally, a conclusion can be drawn from the experiment that TPM4 is highly expressed in hepatoma cells compared with normal hepatic tissues. It is also affiliated with clinicopathological parameters of HCC, from which we can infer TPM4 can serve as a specific biological marker to diagnose HCC.

## Figures and Tables

**Table 1:** Relationship of TPM4 expression between hepatic tissues and hepatocellular carcinoma tissues

TPM4 staining	All cases (%)	Negative expression (%)	Positive expression (%)	*P* value
Hepatocellular carcinoma tissues	110	53(48.2%)	57(51.8%)	0.011
Normal hepatic tissues	10	9(90.0%)	1(10.0%)	

**Table 2:** Relationship of TPM4 expression and clinicopathological features in hepatocellular carcinoma

Variables	TPM4 staining			
	Negative (%)	Positive (%)	Total	*P* value^b^
**Sex**				
Male	44	42	86	0.236
Female	9	15	24	
**Age (year)**				
≤49.1^a^	23	27	50	0.676
>49.1	30	30	60	
**pT Status**				
T1	1 (100.0%)	0 (0.0%)	1	0.000
T2	35 (68.6%)	16 (31.4%)	51	
T3	17 (30.9%)	38 (69.1%)	55	
T4	0 (0.0%)	3 (100.0%)	3	
**Grade**				
1	0 (0.0%)	2 (100.0%)	2	0.015
2	24 (38.1%)	39 (61.9%)	63	
3	21 (60.0%)	14 (40.0%)	35	
4	8 (80.0%)	2 (20.0%)	10	
**Stage**				
I	1 (100.0%)	0 (0.0%)	1	0.000
II	35 (68.6%)	16 (31.4%)	51	
III	16 (29.1%)	39 (70.9%)	55	
IV	1 (33.3%)	2 (66.7%)	3	

a: Mean age; b:* P* values are from Chi-square test.
